# Fine Specificity and Molecular Competition in SLAM Family Receptor Signalling

**DOI:** 10.1371/journal.pone.0092184

**Published:** 2014-03-18

**Authors:** Timothy J. Wilson, Lee I. Garner, Clive Metcalfe, Elliott King, Stefanie Margraf, Marion H. Brown

**Affiliations:** Sir William Dunn School of Pathology, University of Oxford, Oxford, United Kingdom; INSERM- CNRS- Univ. Méditerranée, France

## Abstract

SLAM family receptors regulate activation and inhibition in immunity through recruitment of activating and inhibitory SH2 domain containing proteins to immunoreceptor tyrosine based switch motifs (ITSMs). Binding of the adaptors, SAP and EAT-2 to ITSMs in the cytoplasmic regions of SLAM family receptors is important for activation. We analysed the fine specificity of SLAM family receptor phosphorylated ITSMs and the conserved tyrosine motif in EAT-2 for SH2 domain containing signalling proteins. Consistent with the literature describing dependence of CRACC (SLAMF7) on EAT-2, CRACC bound EAT-2 (K_D_ = 0.003 μM) with approximately 2 orders of magnitude greater affinity than SAP (K_D_ = 0.44 μM). RNA interference in cytotoxicity assays in NK92 cells showed dependence of CRACC on SAP in addition to EAT-2, indicating selectivity of SAP and EAT-2 may depend on the relative concentrations of the two adaptors. The concentration of SAP was four fold higher than EAT-2 in NK92 cells. Compared with SAP, the significance of EAT-2 recruitment and its downstream effectors are not well characterised. We identified PLCγ1 and PLCγ2 as principal binding partners for the EAT-2 tail. Both PLCγ1 and PLCγ2 are functionally important for cytotoxicity in NK92 cells through CD244 (SLAMF4), NTB-A (SLAMF6) and CRACC. Comparison of the specificity of SH2 domains from activating and inhibitory signalling mediators revealed a hierarchy of affinities for CD244 (SLAMF4) ITSMs. While binding of phosphatase SH2 domains to individual ITSMs of CD244 was weak compared with SAP or EAT-2, binding of tandem SH2 domains of SHP-2 to longer peptides containing tandem phosphorylated ITSMs in human CD244 increased the affinity ten fold. The concentration of the tyrosine phosphatase, SHP-2 was in the order of a magnitude higher than the adaptors, SAP and EAT-2. These data demonstrate a mechanism for direct recruitment of phosphatases in inhibitory signalling by ITSMs, while explaining competitive dominance of SAP and EAT-2.

## Introduction

The balance between activation and inhibition in an immune response is critically dependent on the specificity of phosphorylated tyrosine motifs in the cytoplasmic regions of leukocyte surface receptors and associated adaptors for recruitment of intracellular enzymes. The division between activation and inhibition is commonly delineated by specificity of Immunoreceptor Tyrosine based Activation Motifs (ITAMs) and Immunoreceptor Tyrosine based Inhibitory motifs (ITIMs) for an activating kinase or an inhibitory phosphatase respectively. For example, the T cell receptor complex containing multiple ITAMs is an activating receptor and SIRPα with multiple ITIMs is inhibitory. The balance between activation and inhibition is then dependent on recruitment of the receptors at the cell surface.

**Figure 1:**
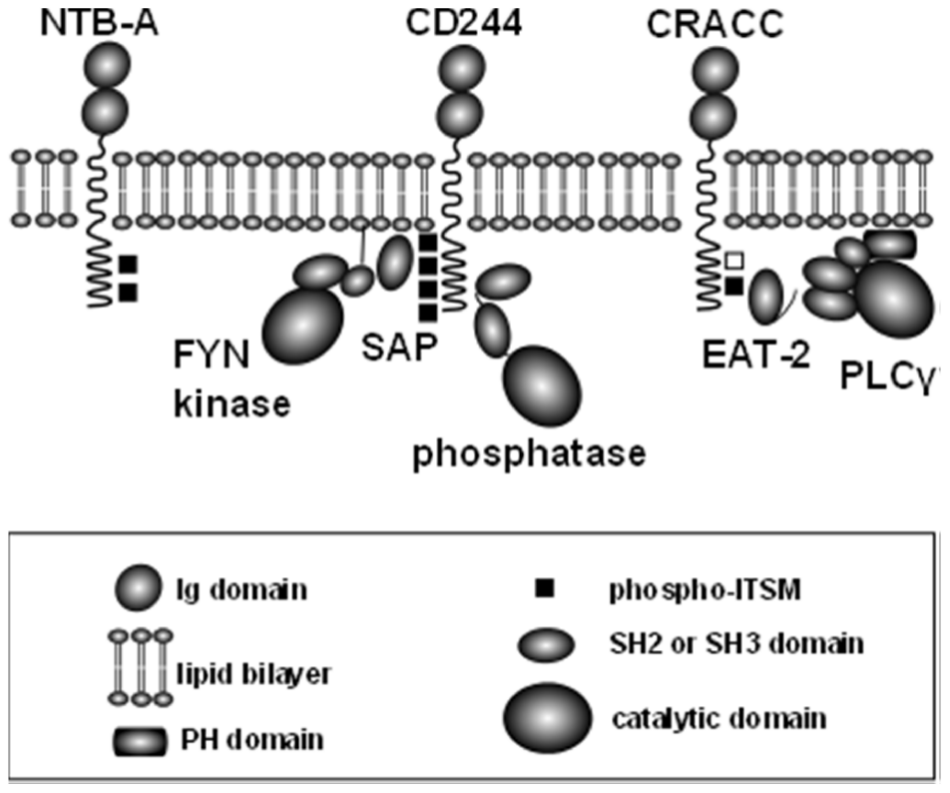
Components implicated in membrane proximal signalling of SLAM-family receptors via ITSMs. CD244, NTB-A and CRACC are depicted because they are expressed on NK92 cells. The adaptor proteins, SAP and EAT2 compete with SH2 domain-containing phosphatases for direct binding to phosphorylated ITSMs (black rectangle). SAP can then recruit FYN which is important for activation via SLAM family receptors. The tail of EAT-2 binds the SH2 domains of PLCγ, providing an additional activation pathway. The ITSM-like motif in CRACC which does not bind SAP or EAT-2 is shown as an unfilled rectangle. SH3 domains are smaller than SH2 domains (ovals). The PH domain in PLCγ is depicted as a shaded rectangle.

An alternative strategy to regulating the balance between activation and inhibition through tyrosine phosphorylation is in the specificity of receptor motifs for the intracellular binding partners. The SLAM family receptors within the CD2 family of leukocyte surface receptors contain multiple Immunoreceptor Tyrosine-based “Switch” Motifs (ITSMs) (Figure 1). These motifs carry the consensus sequence TxYxxV/I/L and biochemical and functional analysis indicate they have overlapping specificity for activating and inhibitory binding partners. CD244 has the greatest number of ITSMs, four (Figure 1). The primary binding partners for these ITSMs are the small SH2 domain containing adapter proteins SAP [1]–[8] and EAT-2 [9]–[11]. *In vivo* and *in vitro* studies in mouse and human have shown that activation through SLAM family receptors is dependent on recruitment of SAP and EAT-2. In the absence of SAP and EAT-2, there is loss of activating function through SLAM family receptors, most notably in humans with mutations in the *SH2D1A* (SAP) gene who develop X-linked lymphoproliferative disease (XLP), a condition characterized by the failure to clear persistent viral infections such as EBV, lymphoid hyperplasia, and lymphoma development [8], [12]. Among the defects in lymphocyte function are impairment of cytotoxic activity in both NK cells and CD8+ T cells [13]–[18].

NK cells express both SAP and EAT-2 proteins [5], [9]. The best characterized molecular interaction of SAP family adapters with an intracellular enzyme is the recruitment of the Src-family kinase Fyn (Figure 1) [19]–[23]. There is evidence that SAP can also couple SLAM family receptors to other cytosolic effector enzymes [24]–[26] through the conserved interaction of SH3 domains with Arg78 on SAP [20]. EAT-2 lacks a similar structural motif and so cannot interact with SH3 domains. Instead, it contains a conserved YxxV motif which binds SH2 domain containing proteins [22], [27]. In mice, NK cell-mediated cytotoxicity against SLAM-family receptor-expressing target cells is impaired in both SAP^−/−^ and EAT-2/ERT^−/−^ mice, ERT being an additional EAT-2 homologue in mice [21], [28], [29]. Activation through EAT-2 is dependent on tyrosine phosphorylation of the tail of EAT-2 [27], [30]. Recruitment of Fyn by EAT-2 [11], [21], [27] may contribute to early phosphorylation events, however, an interaction between PLCγ1 and the tyrosine phosphorylated tail of EAT-2 [11], [27] suggests a mechanism of recruitment of PLCγ through SLAM family receptors (Figure 1) [9], [11], [27], [31], [32]. Furthermore, mouse NK cells lacking PLCγ2 fail to mobilize calcium and have impaired cytotoxicity through all activating receptors examined including CD244 [33]. PLCγ1 deficient mouse embryos are not viable [34] but complementation with PLCγ1 indicated specific niches for each of the two isoforms [35].

In addition to loss of activating function in the absence of SAP, EAT-2 and ERT, SLAM family receptors exhibited effects which suggested the receptors were actively attenuating signalling [28], [36]. Recently signal attenuation by Ly108/NTB-A has been dissected providing evidence for biochemical loops of activation and inhibition [23], [37], and analysis of functional dependence on the tyrosine phosphatases, SHP-1 and SHP-2 versus an inositol phosphatase, SHIP suggested a dominant role for the latter [23]. Colocalisation of SHP-1 with a SLAM family receptor in the absence of SAP indicates a role for the tyrosine phosphatases in signal attenuation [37], [38]. Physical associations with inhibitory binding partners of SLAM family receptor ITSMs have been proposed based on indirect assays such as coprecipitation [5], [18], pulldowns [39] and colocalization, [37], [38] and on direct binding studies on protein arrays [40] and surface plasmon resonance (SPR) [22], [40].

We sought to more clearly define the hierarchy of binding partners for ITSMs and the EAT-2 tail. We distinguished between specificity of ITSMs for the adaptors, SAP and EAT-2 and for inhibitory binding partners [22], [27], [39], [40]. PLCγ1 and PLCγ2 were identified as binding partners for the EAT-2 tail, and functional experiments confirmed that SLAM family receptor mediated killing in human is critically dependent on SAP, PLCγ1 and PLCγ2 and to a lesser extent on the presence of EAT-2.

## Materials and Methods

### Peptides and recombinant proteins

Synthetic biotinylated phosphopeptides were obtained from Peptide Protein Research Ltd. The peptide sequences from human proteins are: LAT Y132 (Biotin-HNPGpYLVVLPD); EAT-2 Y127 (Biotin-NSNSDpYVDVLP); and CD244 ITSM1 (Biotin-EFLTIpYEDVKD); CD244 ITSM1+ITSM2 (Biotin-EFLTIpYEDVKDLKTRRNHEQEQTFPGGGSTIpYSMIQS; CD244 ITSM3+ITSM4 (Biotin-PAYTLpYSLIQPSRKSGSRKRNHSPSFNSTIpYEVIGK); SIRPα ITIM1+ITIM2 (Biotin-TNDITpYADLNLPKGKKPAPQAAEPNNHTEpYASIQT); SHIP-1 Y865 (Biotin-TREKLpYDFVKT); CRACC ITSM1 (Biotin-GENTEpYDTIPH) and CRACC ITSM2 (Biotin-PANTVpYSTVEI) and as published previously [22], [27]. Recombinant human proteins, SAP and EAT-2 [22], SHIP SH2 from SHIP-1 [41] and SHP-2 N-terminal + C-terminal (N+C) SH2 domains (Oxford Protein Production Facility, OPPF catalogue number 3911; aa 2–219, EC = 23,045 M^−1^ cm^−1^) were produced in house and at the Oxford Protein Production facility. Biotinylated EAT-2 was produced by expression of full length human EAT-2 with a C-terminal BirA tag (NSGSLHHILDAQKMVWNHR) in the E coli expression vector pTrcHisA. Constructs encoding the 6xHis-tagged tandem SH2 domains plus the SH3 domains of PLCγ1 (Swiss-Prot: P19174, aa 536–863) and PLCγ2 (Swiss-Prot: P16885, aa 519–838) were generated in pTrcHisA (5′ joining sequence with BglII site underlined: gag atc tcc translates as EIS). The molecular weight (MW) and theoretical extinction coefficients (EC) of recombinant PLCγ1 and PLCγ2 SH2-SH2-SH3 domains were PLCγ1; MW = 42531, EC = 63,830 M^−1^ cm^−1^ and PLCγ2; MW = 42415, EC = 67,270 M^−1^ cm^−1^.

### Protein expression and purification

Soluble recombinant proteins were produced in BL21(DE3)pLysS E. coli (Calbiochem) at 37°C in 2TY medium and induced at OD 600 = 0.6 with 0.1 mM IPTG for 4 h or in 2XYT auto-induction (Formedium) medium overnight. Bacterial pellets were resuspended in ∼0.1 volume 10 mM HEPES pH7.4, 150 mM NaCl and used immediately or stored at −20°C in 10 ml aliquots. His-tag mediated protein purification was performed on a Co^2+^ or Ni^2+^ column by standard protocols. Protein purification of SAP, EAT-2, PLCγ1 and PLCγ2 was optimized by being performed on the day of SPR experiment in order to use protein at the highest activity with minimum aggregation. Where applicable, proteins were stored frozen in 10–25 mM HEPES pH 7.4, 200–300 mM NaCl (pH 7.4).All proteins were purified by size exclusion chromatography immediately prior to SPR analysis.

### Protein pull-down and LC-mass spectrometry

For pull-down of associated proteins, streptavidin Sepharose (GE Healthcare) was pre-coated with saturating amounts of peptide or EAT-2. NK92 cells (4×10^8^) were treated with 500 μM pervanadate for 15 minutes, then lysed in PBS, 1% Triton X-100, 1 mM sodium orthovanadate in the presence of mammalian protease inhibitor solution (Sigma). Lysates were pre-cleared by centrifugation, followed by incubation with streptavidin Sepharose. Peptide or EAT-2-coated streptavidin beads were incubated with NK92 lysates for 2 hours at 4°C with rotation. Beads were washed once with 15 ml ice cold PBS/1% (v/v) Triton X-100. Bound protein was eluted using 100 mM glycine-HCl pH 2.5.

Protein samples were prepared for mass spectrometry as described previously [42]. Briefly, eluted proteins were spun onto the membranes of Vivacon 500 filters (10,000 MWCO – Sartorius). Membranes were washed once with PBS then proteins were denatured with 8 M urea, reduced with 10 mM DTT in 25 mM ammonium bicarbonate buffer, followed by alkylation with 50 mM iodoacetamide. Proteins were then digested overnight with trypsin. Tryptic peptides were then eluted from the membrane and analyzed on an Ultimate 3000 HPLC-coupled Orbitrap XL mass spectrometer. Proteins were identified by liquid chromatography-coupled tandem mass-spectrometry (LC-MS/MS). Raw data files were converted to mzXML format using ReADW (v. 4.2.1) and submitted to the Central Proteomics Facilities Pipeline [43] for database comparison and peptide identification. Searching against the International Protein Index (IPI) human protein sequence database for protein identification [44], protein and peptide hits were statistically validated by applying a 1% false discovery rate (FDR) by searching against a concatenated target/decoy database. Datasets were searched with variable peptide modifications for phosphotyrosine, phosphoserine, phosphothreonine, oxidation of methionine and deamination.

Protein identification data were exported into ProteinCenter (v3.8.3013 – Thermo Fisher) for filtering and annotation. Results were filtered to remove any proteins with less than 3 unique peptides, less than 10 percent sequence coverage, or median protein probability scores less than 0.5. To establish sample specificity, any protein with at least 10-fold greater sequence coverage in one sample compared with the other was considered to be specific for that sample.

### Surface plasmon resonance (SPR)

Measurements of protein-protein affinity were performed on BIAcore 3000 and T200 systems using CM5 or biotin capture chips (GE Healthcare Ltd, UK). Streptavidin was coupled to CM5 sensor chips using standard NHS/EDC chemical linkage, followed by immobilization of biotinylated peptides. The levels of peptide immobilized were approximately 25 and 50 response units respectively of single and tandem tyrosine motifs to facilitate saturating binding and limitation of “bridging” [22], [45]. Purified recombinant proteins were passed over the immobilized peptides at a range of concentrations in HBS-EP buffer, 10 mM HEPES pH 7.4, 150 mM NaCl, 3.4 mM EDTA, 0.005% surfactant, P20. Equilibrium binding coefficients and dissociation and association rates were calculated using BIAevaluation and/or GraphPad Prism 5.4 software.

### Cell culture

Mouse P815 cells (ATCC #TIB-64) and the human B cell line, 721.221 [46] were maintained in complete RPMI (RPMI 1640/10% Fetal Calf Serum (FCS) with supplemental sodium pyruvate, L-glutamine, non-essential amino acids, and kanamycin). NK92 cells (ATCC #CRL-2407) were obtained from Marco Colonna (Washington University) and maintained in complete RPMI plus 1000 U/ml recombinant human IL-2, generously provided by Marco Colonna and Gillian Griffiths (University of Cambridge). 293 T cells (ATCC #CRL-3216) were cultured and transfected in DMEM/10% FCS with L-glutamine, sodium pyruvate, and kanamycin.

### RNA interference

Lentiviral constructs expressing gene-specific shRNAs in vector pLKO.1 were obtained from Open Biosystems. Panels of 5 shRNAs were tested for each protein to select the most effective construct. The specific clones used were as follows: TRCN0000006979 for PLCγ1, TRCN0000002322 for PLCγ2, TRCN0000082710 for SAP, and TRCN0000082657 for EAT-2. As a non-targeting control, the hairpin sequence 5′-GCGCGCTTTGTAGGATTCGTTCAAGAGACGAATCCTACAAAGCGCGCTTTTTT was expressed in pLKO.1. Lentiviral particles were produced through polyethylenimine (PEI-Sigma) co-transfection of 293 T cells with pLKO.1 along with packaging vectors pMD2.G and pSPAX2 (Addgene; Didier Trono Laboratory). Virus was produced in RPMI 1640/10% heat-inactivated FCS for 48–72 hours. Viral supernatants were collected and NK92 cells were transduced twice overnight in the presence of 1000 U/ml IL-2. Cells with stable shRNA expression were selected using 3 μg/ml puromycin (Invitrogen) for 72 hours prior to use in functional assays.

### Quantitation of protein expression

For quantitation of protein expression, NK92 cell lysates were prepared using buffer (150 mM NaCl, 20 mM Tris-HCl pH 7.4, 10% glycerol, 2 mM EDTA, 10 mM NaF, 1 mM PMSF) containing 0.5% Triton X-100. Lysates were resolved by SDS-PAGE under reducing conditions using Novex gradient gels and transferred to nitrocellulose membranes for western blotting by standard protocols. Following RNA interference, blots containing protein lysates from 10^5^ cells were developed with: anti-EAT-2 antibody (rabbit polyclonal; a kind gift from Marco Colonna and Ilaria Tassi), anti-PLCγ1 (2822) from Cell Signalling Technology or anti-human PLCγ2 (Q-20) from Santa Cruz. For quantitation of SAP and EAT-2 protein levels in NK92 cells, the equivalent of 0.5−2×10^6^ cells from lysates prepared at 5×10^7^ cells/ml was analysed alongside known amounts of recombinant protein standards, recombinant full-length human SAP (50, 25, 12.5 and 6.25 ng) or EAT-2 (20, 10, 5 and 2.5 ng), generated as previously described and developed with anti-SAP antibody (rabbit polyclonal clone FL128, Santa Cruz Biotechnology) or anti-EAT-2. For chemiluminescent detection, HRP conjugated anti-mouse and anti-rabbit IgG antibodies were obtained from Cell Signaling Technology. SHP-2 levels in 10^5^ cell equivalents were analysed in a similar manner with recombinant SHP-2 N+C as a standard (25, 50 and 100 ng) and anti-SHP-2 (Cell Signaling #3752). Fluorescent detection of protein bands was performed on the LI-COR Odyssey Sa system. IRDye 680LT-conjugated anti-rabbit IgG and IRDye 800 CW anti-mouse IgG were obtained from LI-COR Biosciences. Quantitation of protein expression was performed using LI-COR Odyssey Sa software version 1.0. In initial experiments, estimations of % knockdown in comparison with another protein blotted with a specific antibody or in comparison with a nonspecific “reference” band gave the same result. Subsequently, a “reference” band was routinely used. After RNA interference, SAP expression was also quantified by intracellular staining using anti-human SAP (XLP-1D12) from Cell Signalling Technology and median fluorescence intensity measured by flow cytometry.

### Cytotoxicity assays

Cytotoxicity through individual receptors was measured in a redirected lysis assay. P815 target cells were labeled for 3 hours with EXPRE^35^S^35^S Protein Labeling Mix (Perkin Elmer, Inc.) in cysteine/methionine-free RPMI (Sigma). Target cells were washed 3 times in complete RPMI and dispensed into 96-well round bottom plates at 5000 cells/well. Lentiviral-transduced NK92 cells were harvested and mixed with target cells at the indicated effector:target ratio in duplicate unless otherwise stated in the presence of IL-2. The indicated antibodies were added to the cellular mixture for the duration of the 4 hour cytotoxicity assay. The antibodies used to induce cytotoxicity were as follows: anti-NKp30 (P30.15) and anti-NTB-A (NT-7) were obtained from Biolegend; anti-CRACC (162) and anti-CD58 (MEM-63) were obtained from Abd Serotec; anti-CD2 (X53) and anti-CD244 (C1.7) were purified in our laboratory. The numbers of independent experiments were: SAP – 2, EAT2 – 3, PLCγ1 - 4, and PLCγ2 - 2.

## Results

### SAP and EAT-2 show differential binding to motifs in SLAM family receptors

**Figure 2:**
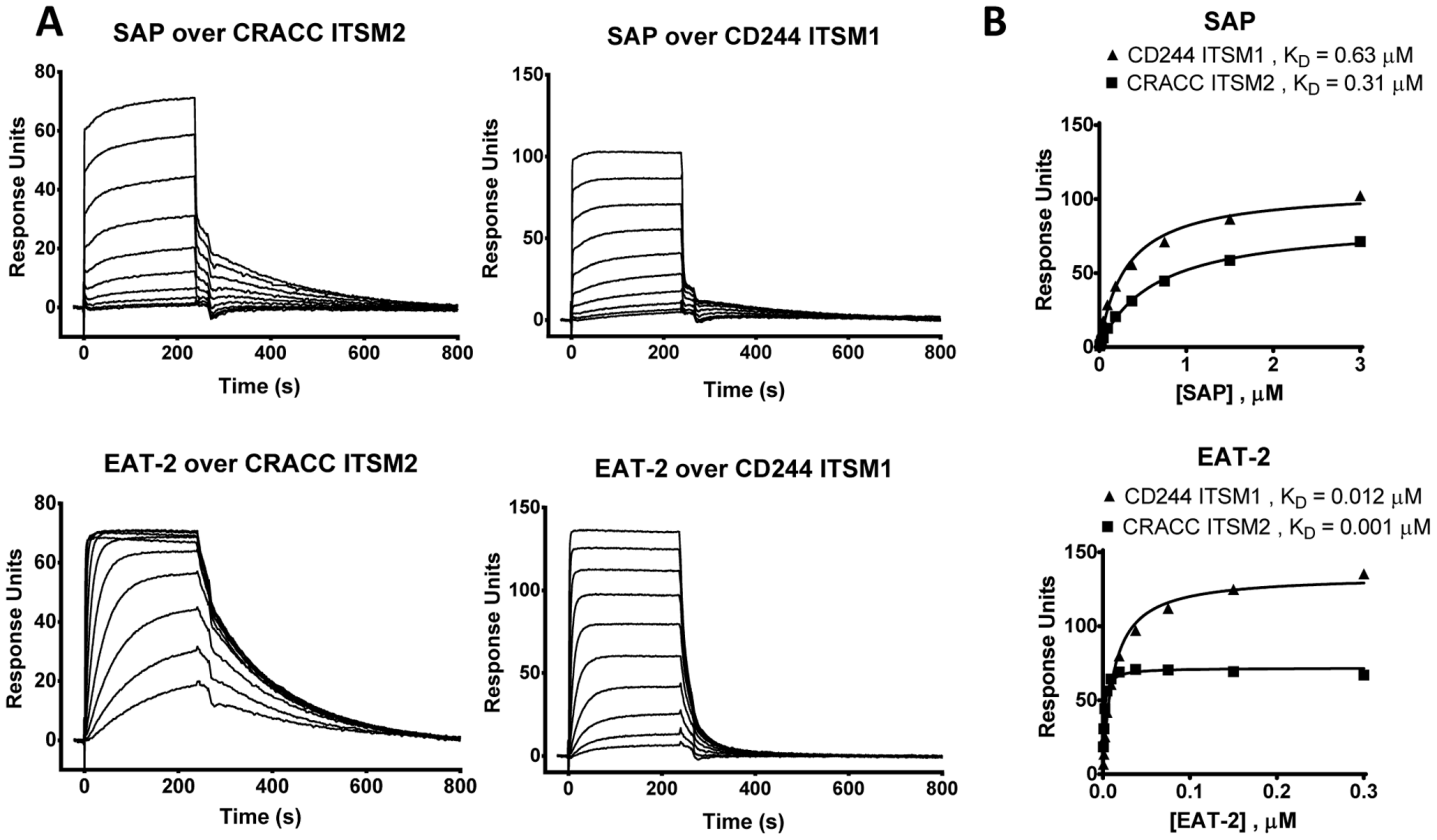
SAP and EAT-2 show differential binding to motifs in SLAM family receptors. Overlaid SPR sensorgrams (A) and equilibrium binding curves (B) for ten serial injections of two-fold dilutions of human full-length SAP (top concentration 3 μM) or EAT-2 (top concentration 0.3 μM) over immobilised phosphorylated peptides representing CRACC ITSM1, CRACC ITSM2 or CD244 ITSM1 at 37°C. Signal from a blank reference cell was subtracted as background. No specific association between CRACC ITSM1 and SAP or EAT-2 was observed (not shown). K_D_ values were determined by fitting equilibrium binding data using a one-site specific binding model. Proteins were purified on the same day as SPR analysis.

The most distinct differences between SAP and EAT-2 are the mechanisms of recruitment of downstream effectors and their distribution. The specificities of their SH2 domains overlap, introducing the concept of competition between them for ITSMs in cells where they are co-expressed, including NK cells. In our previous studies we focused on comparison between human and mouse CD244 ITSMs showing that SAP and EAT-2 had similar specificity for phosphorylated ITSMs in both species [22]. Signalling through the SLAM family receptor, CRACC has been reported to be independent of SAP [9], [30], suggesting a difference in specificity of the CRACC ITSMs for SAP and EAT-2. We measured the affinity of SAP and EAT-2 for CRACC peptides by SPR (Figure 2). Binding of SAP to CRACC ITSM2 was observed, however, EAT-2 bound about a hundred fold more strongly to CRACC ITSM2 with a K_D_ = 0.003 μM (Figure 2 and Table 1). The preference of CD244 ITSM1 for EAT-2 was less striking, with the K_D_ for binding of EAT-2 to CD244 ITSM1 being one order of magnitude stronger than SAP (Figure 2, Table 1). This selectivity of the CRACC ITSM may explain the failure to detect SAP binding to CRACC by immunoprecipitation [9] and the reported dominance of EAT2/ERT in mouse CRACC signalling [30]. Neither SAP nor EAT-2 bound phosphorylated human CRACC ITSM1 (data not shown). In mouse the tyrosine motif (ADYDTI) in the equivalent position to human CRACC ITSM1 deviates from the specificity and consensus for an ITSM (TxYxxV/I/L), did not bind EAT-2 and differed from ITSM2 in its functional effects [30]. These measurements were carried out with full length SAP and EAT-2 purified on the same day (see Methods) and affinities were comparable to those obtained with the isolated SH2 domains ([22] and data not shown). Lower affinities obtained with full length SAP and EAT-2 in our earlier study [22] were most likely related to protein activity.

**Table 1:** Equilibrium dissociation constants for activating proximal binding partners in SLAM family receptor signal transduction.

Peptide:	SAP			EAT-2		
	K_D_	range	*n*	K_D_	range	*n*
CRACC ITSM2	0.44	0.19–0.67	*8*	0.003	0.001–0.007	*8*
CD244 ITSM1	0.18	0.11–0.31	*4*	0.02	0.01–0.03	*4*

Mean K_D_ values (μM) measured at 37°C for soluble recombinant proteins binding to immobilised phosphorylated peptides by SPR, range and number (n) of independent measurements. Proteins were purified on the same day as SPR analysis.

### SAP can regulate signalling through CRACC

**Figure 3:**
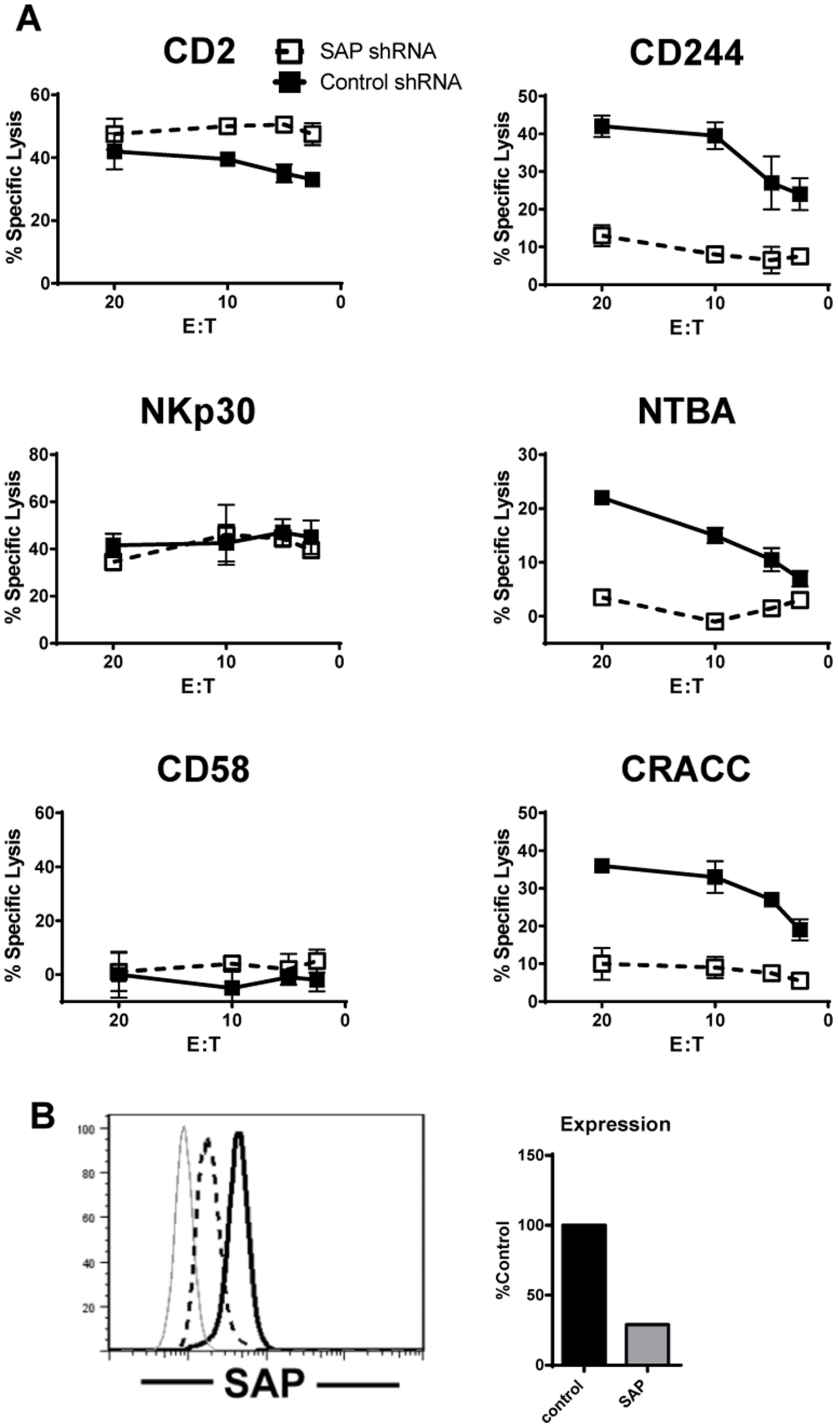
SAP promotes cytotoxicity through human SLAM family receptors including CRACC. A) Redirected lysis of P815 cells by NK92 cells expressing control or SAP-specific shRNA using the indicated activating antibodies. Error bars represent the mean +/− SEM of duplicate wells. B) Intracellular SAP expression in NK92 cells was analysed by flow cytometry. After subtraction of isotype control (grey line), the % median fluorescent intensity of SAP expression in SAP knockdown (dashed line) compared with control (black line) NK92 cells is shown in the bar graph. Data are representative of 2 independent experiments. E:T = effector:target.

Overlapping specificity of SAP and EAT-2 for ITSMs suggested both SAP and EAT-2 had the potential to regulate signalling through SLAM family receptors, including CRACC. We tested for functional dependence of cytotoxicity on SAP and EAT-2 in a human NK cell line, NK92, targeting expression by lentiviral transduction of a specific shRNA. Receptor-specific effects on cytotoxicity were determined by redirected lysis assays using the Fc receptor-expressing, mouse mastocytoma cell line P815 as targets. Antibodies to CD244, NTB-A, and CRACC, the SLAM family receptors expressed on NK92 cells were used to cross-link these receptors and anti-CD58 was used as a negative control. To examine the specificity of the relative contribution of proteins important for SLAM family activating signal transduction in additional receptor pathways, the activation of cytotoxicity through CD2 (which contains multiple PxxP motifs for SH3 domain binding) and NKp30 (which associates with CD3ζ) were tested. The cytotoxic activity of the SLAM family receptors, including CRACC, was reproducibly reduced (n = 2) following SAP knockdown (Figure 3A, B). Interestingly, the reduction in SAP expression consistently resulted in higher CD2 mediated cytotoxicity, indicating possible interference by SAP in unknown pathways downstream of CD2.

**Figure 4:**
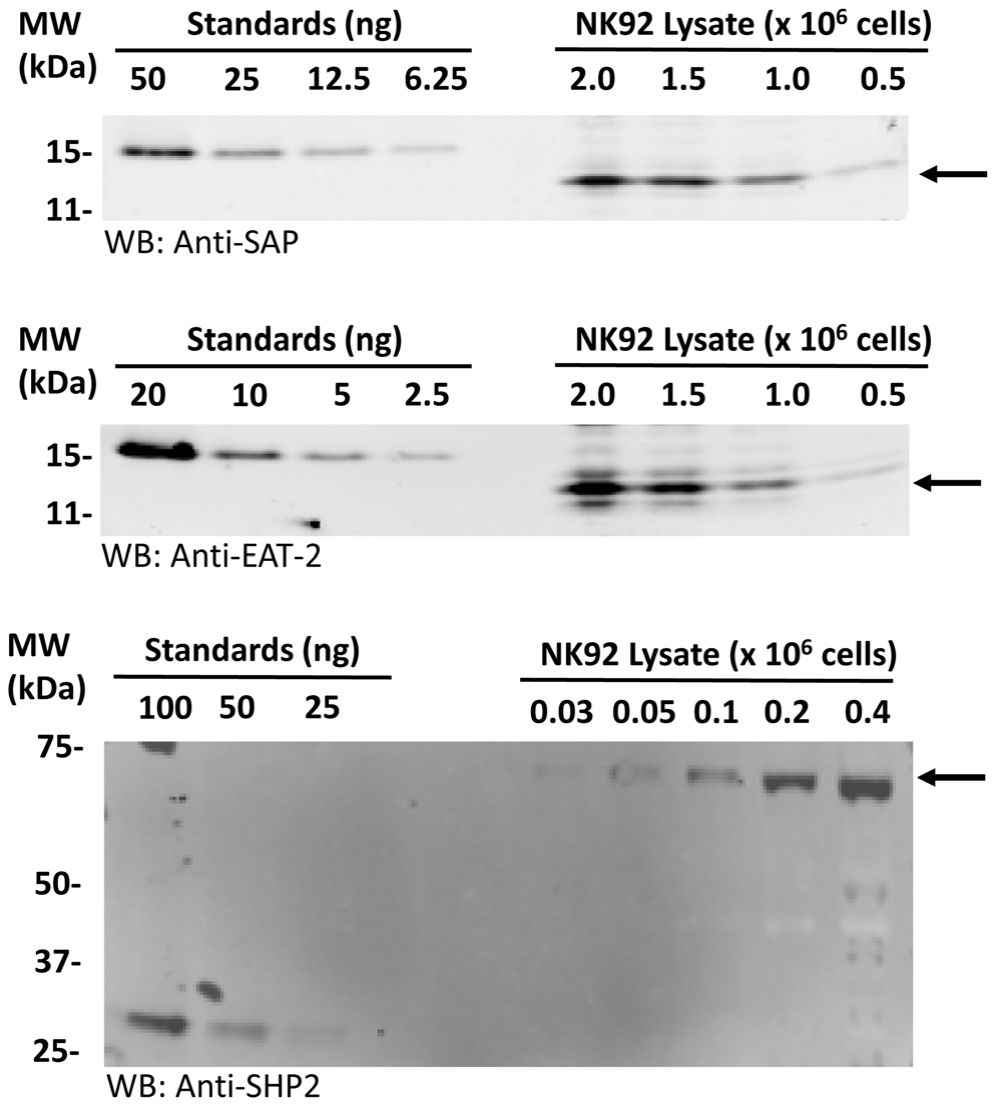
The concentrations of SAP, EAT-2 and SHP-2 in NK92 cells. Recombinant SAP, EAT-2, SHP-2 N+C and NK92 cell lysates were analysed by Western blotting using the LI-COR Odyssey Sa system. A representative blot for each protein is shown. 1×10^6^ NK92 cells express 30 ng of SAP and 7 ng of EAT-2. 0.1×10^6^ NK92 cells express 92 ng of SHP-2. One representative blot of each protein is shown.

Since there is competition between EAT-2 and SAP for ITSMs, the concentrations of SAP and EAT-2 are factors in determining dependence of SLAM family receptors on these adaptors. We measured the concentration of SAP and EAT-2 in NK92 cells by western blotting (Figure 4, Table 2) and determined a SAP to EAT-2 ratio of 4∶1. Using an approximation of the diameter of NK92 cells as 10 μm, which gives a cellular volume of 523 fL, we estimated the total cellular concentration of SAP as 1.9 μM and EAT-2 as 0.44 μM.

**Table 2:** Quantitation of SAP, EAT-2 and SHP-2 in NK92 cells.

	SAP	EAT-2	SHP-2
Mass (ng)/10^6^ cells	20, 30, 33	5, 8, 9	928, 787
Mean (ng)/10^6^ cells	28	7	858
S.E.	4	1	71
Concentration μM	1.9	0.44	24
*n*	3	3	2

Protein mass (ng) detected in lysate 10^6^ NK92 cells and estimated concentration per cell are shown. n =  number of independent experiments.

**Figure 5:**
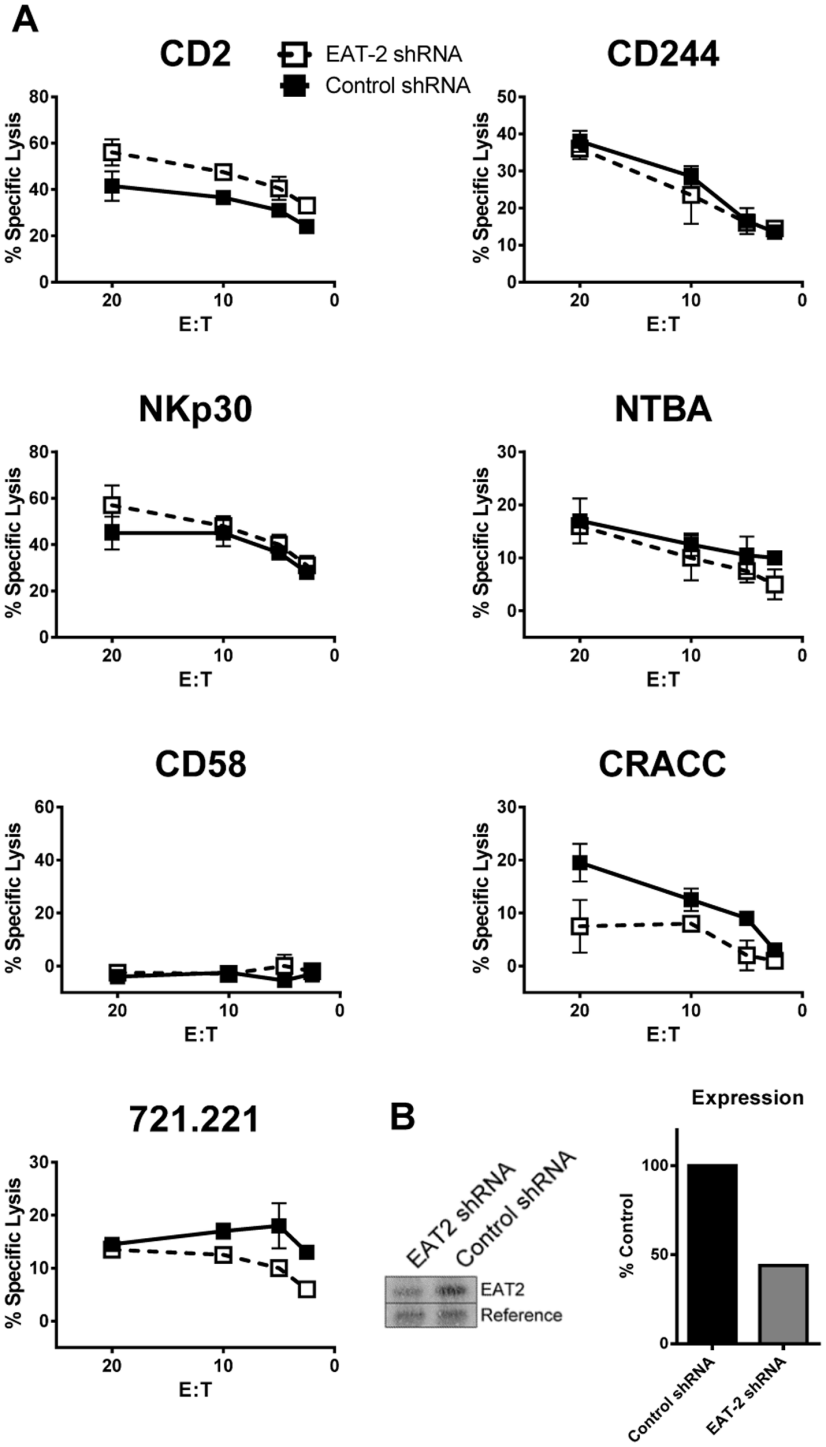
EAT-2 knockdown has minimal effects on NK92 cytotoxicity. A) Redirected lysis of P815 cells by NK92 cells expressing control or EAT2-specific shRNA using the indicated activating antibodies, or natural cytotoxicity against the human B cell line 721.221. B) Quantitation of EAT-2 expression in NK92 cells analysed by western blotting is shown in the bar graph. Error bars represent the mean +/− SEM of duplicate wells. Data are representative of 3 independent experiments. E:T = effector:target.

RNA interference in the redirected lysis assay in NK92 cells reduced the concentration of EAT-2 by up to ∼50% (Figure 5). In the majority of experiments (n = 3), no effect of EAT-2 knockdown on SLAM family mediated cytotoxicity was observed. This may be due to an insufficient reduction in intracellular EAT-2 concentration. We determined the intracellular EAT-2 concentration in NK92 cells to be 0.44 μM, and so a 50% reduction in EAT-2 through shRNA mediated knockdown will yield an intracellular concentration of approximately 0.2 μM. Based on the dissociation constants for EAT-2 binding to CD244 and CRACC (Figure 2), an EAT-2 concentration of 0.2 μM would approach saturation of the phosphorylated ITSMs. This is in contrast to SAP where the endogenous concentration, 1.9 μM was knocked down to 0.6 μM. According to the equilibrium binding data (Figure 2), this concentration of SAP is well below saturation for both CD244 and CRACC, and so we observe a functional impact of the knockdown. In each experiment CD2 and NKp30 mediated cytotoxicity were consistently enhanced by EAT-2 knockdown (Figure 5 and data not shown). Analysis of spontaneous lysis of the NK-susceptible target B cell line 721.221 indicated that engagement of multiple receptors facilitates the activating function of EAT-2 in NK92 cells ((Figure 5 [39]).

### ITSMs show a hierarchy in binding SH2 domains from activating and inhibitory signalling proteins

Analysis of binding of SAP and EAT-2 to ITSMs revealed differences in specificity which can explain different modes of activation. Similarly, differences in specificity of inhibitory SH2 domain containing proteins for ITSMs are relevant to understanding mechanisms of inhibition by ITSMs. We focused on CD244 ITSMs to establish general paradigms. As CD244 binding to the adaptors, SAP and EAT-2 is dependent on phosphorylation [11], [22], [47] only binding to phosphorylated peptides was analysed. We first confirmed the potential of CD244 ITSM1 to recruit activating and inhibitory binding partners by performing pull-down experiments with human NK cell lysates and mass spectrometry analysis of proteins bound specifically to CD244 ITSM1 when compared with a control phosphopeptide (SHIP-1 Y_865_). In addition to identifying SAP and EAT-2, the SH2 domain containing phosphatases SHIP-1, SHIP-2, SHP-1, SHP-2 and CSK, as well as GRB-2 and PLCγ1 were also pulled down by the ITSM peptide (Table 3).

**Table 3:** Identification of proteins interacting with CD244 ITSM1.

Protein	CD244 ITSM1 Unique Peptides	Control pTyr Unique Peptides	CD244% Coverage	Control % Coverage
SAP	7	1	74	11
SHIP-1	51	1	53	2
SHP-1	28	0	50	0
SHP-2	25	0	45	0
c-Src Tyrosine Kinase (CSK)	11	0	36	0
EAT-2	3	0	34	0
Growth factor receptor-bound protein 2 (GRB2)	3	0	25	0
SHIP-2	19	0	21	0
Phospholipase C – gamma 1	10	0	15	0

SH2 domain containing proteins identified by LC-MS/MS that associated with the phosphorylated CD244 ITSM1 are listed compared with a control phosphopeptide derived from SHIP-1 Y_865_.

**Figure 6:**
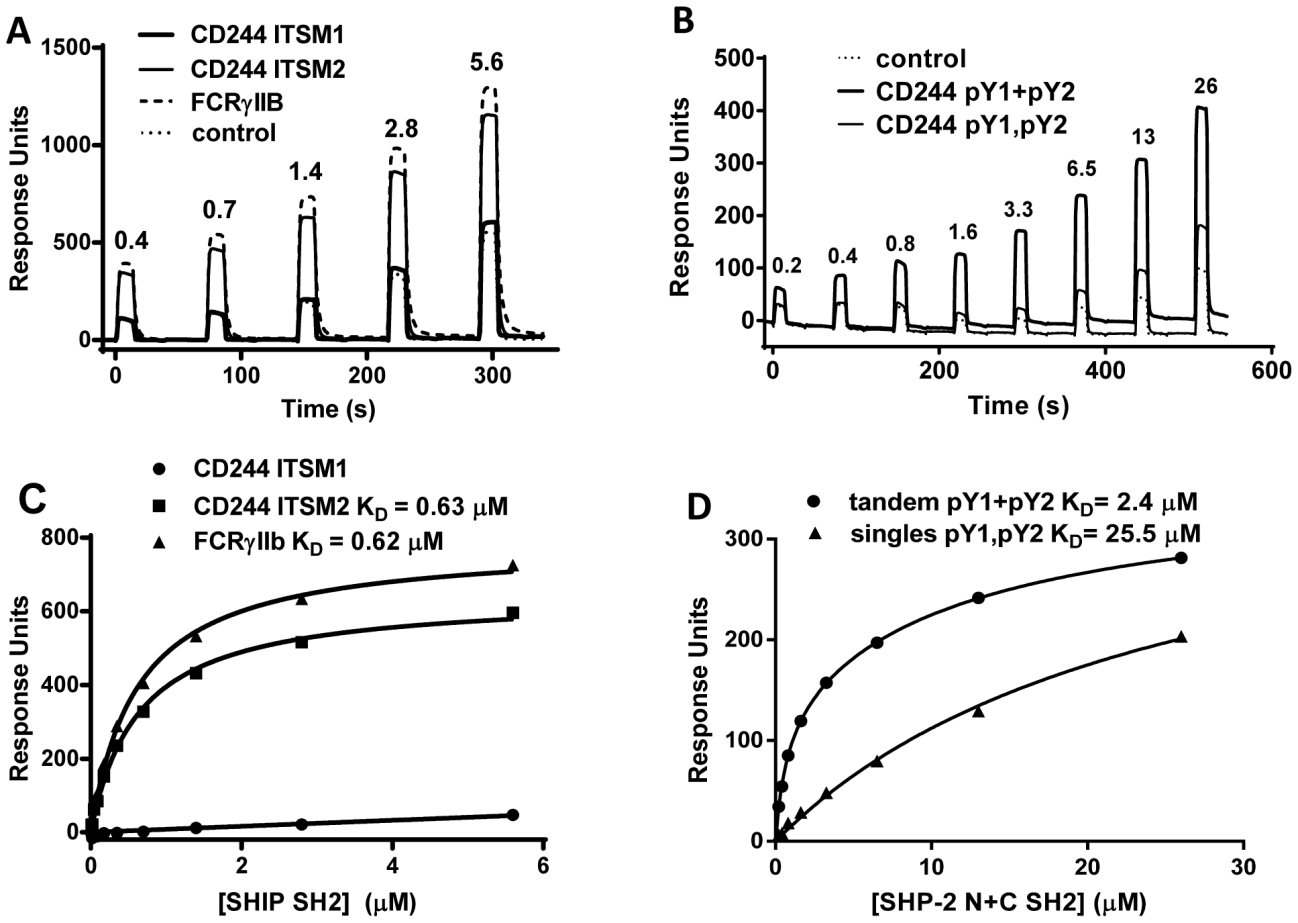
Specificity of SH2 domain-containing phosphatases in SLAM family receptor signal transduction. SPR sensograms of increasing concentrations (μM) of hSHIP SH2 (A) and SHP2 N+C SH2 (B) injected over immobilised phosphorylated peptides at 37°C. K_D_ values were determined by fitting (solid lines) equilibrium binding (symbols). (A, C) SHIP SH2 domain binding to CD244 single ITSMs. (B, D) Increased affinity of SHP2 tandem SH2 domains for tandem ITSMs compared with the mixture of single ITSMs.

We examined whether hierarchies of specificity of CD244 ITSMs for activating and inhibitory binding partners differ. SHIP-1 was studied as an example of a single SH2 domain containing phosphatase and SHP-2 an example of tandem SH2 domains. We compared the relative specificity of the single SH2 domain from SHIP-1 for the 4 ITSMs from CD244 (Figure 6A, C and Table 4). A representative sensorgram trace for binding of SHIP-1 SH2 domain to CD244 ITSM1 and ITSM2 and a positive control, a well characterized SHIP binding peptide from FCRγIIb, are shown (Figure 6A). Equilibrium binding analysis (Figure 6C, Table 4) revealed a preference of SHIP SH2 domain for CD244 ITSM2 and ITSM3. The same pattern was observed in binding studies with mouse SHIP SH2 domain and mouse CD244 ITSMs (data not shown). While the pattern of binding to the different ITSMs was similar for the inhibitory SHIP and activating adaptor domains (Table 4), SAP and EAT-2 consistently bound ITSMs ten to a hundred fold more strongly than SHIP-1. FCRγIIb was included as a monitor of SHIP SH2 domain activity and the K_D_ was comparable with previous measurements [41].

**Table 4:** Equilibrium dissociation constants for inhibitory proximal binding partners in SLAM family receptor signal transduction.

	SHIP (SH2)			SAP			EAT-2		
Peptide:	K_D_	range	*n*	K_D_	range	*n*	K_D_	range	*n*
CD244 ITSM1	11	11–12	*3*	0.5	0.3–0.7	*5*	0.06	0.01–0.14	*5*
CD244 ITSM2	2.3	0.6–6	*8*	0.02	0.01–0.02	*2*	0.06	0.02–0.15	*3*
CD244 ITSM3	2.2	0.5–4	*7*	0.08	0.05–0.12	*3*	0.07	0.04–0.13	*3*
CD244 ITSM4	5.6	0.4–17	*5*	1.9	1.2–2.7	*2*	0.04	0.02–0.06	*3*
FCRγIIb (CD32)	0.47	0.1–0.8	*13*	–	–	*–*	–	*–*	–

	SHP-2 (SH2-SH2) 37°C			SHP-2 (SH2-SH2) 25°C		
Peptide:	K_D_	range	*n*	K_D_	range	*n*
CD244 ITSM1	29	22–34	*4*	–	–	–
CD244 ITSM2	24	–	*1*	–	–	–
CD244 ITSM1,ITSM2*	22	6–27	*5*	–	–	–
CD244 ITSM1+ITSM2	2.2	0.8–3.2	*8*	1.4	0.4–1.2	*6*
CD244 ITSM3+ITSM4	10	8–12	*2*	7.4	5–10	*8*
SIRPα ITIM1+ITIM2	1.0	0.4–3.3	*4*	0.5	0.1–0.7	*6*
SIRPα ITIM3+ITIM4	–	–	*–*	6.4	6–10	*5*

Mean K_D_ values (μM) at 37°C for soluble recombinant proteins binding to immobilised phosphorylated peptides by SPR, range and number (n) of independent measurements. *Equimolar mixture of single ITSM on chip.

### Tandem SH2 domains bind tandem ITSMs and ITIMs

Binding of inhibitory tyrosine phosphatases through their SH2 domains to ITIMs is enhanced by phosphorylation of two tyrosine motifs which are appropriately spaced [45]. We identified pairs of ITSMs (based on spacings of 23-30 amino acids between tyrosines) in the cytoplasmic tails of SLAM family receptors including human CD244 ITSMs 1 and 2 and human CD244 ITSMs 3 and 4 with the potential to simultaneously bind to tandem SH2 domains of the tyrosine phosphatases. We measured binding of SHP-2 tandem SH2 domains (recombinant SHP-1 SH2 domains were unstable in solution) to tandem ITSMs in CD244 (Figure 6B and Table 4). A representative sensorgram (Fig. 4B) shows that equilibrium binding was achieved with minimal interference with aggregated material only apparent at the higher concentrations of soluble SHP-2. We measured an order of magnitude increase in affinity of binding of tandem SH2 domains to the tandem ITSM peptides compared with individual ITSMs (Fig. 4D, Table 4). No increase in affinity was observed when both individual ITSM peptides were immobilized. Binding to the tandem peptide representing CD244 ITSM3+ITSM4 was 3-5 fold weaker compared with CD244 ITSM1+ITSM2 at 37°C and 25°C and we observed the same phenomena for the SIRPα tandem ITIM peptides (Table 4 and data not shown).

Binding of SHP-2 tandem SH2 domains to CD244 was two-three fold weaker, but comparable with binding to tandem ITIMs from a well characterized inhibitory receptor SIRPα [48] (Figure 6D, Table 4). We obtained similar K_D_ values for at least three different preparations of SHP-2 tandem SH2 domains and observed similar affinities with full length SHP-2 (not shown). Quantitation of the levels of SHP-2 in NK92 cells, as was done for SAP and EAT-2, indicated there is an order of magnitude higher concentration compared with SAP, and is approximately an order of magnitude higher than the K_D_, indicating that SHP-2 will be competitive for binding highly phosphorylated receptor.

### The tail of EAT-2 binds phospholipase C (PLC) γ1 and PLCγ2

In contrast to the characterization of the specificity of ITSMs, the underlying molecular basis of activation and inhibition by EAT-2 has not been clarified. Activating effects of EAT-2 downstream of SLAM family receptors are dependent on phosphorylation of the tyrosine motif in the tail [27], [29]. In our previous studies, PLCγ1 was identified as a potential binding partner for the EAT-2 tail in human T cell blasts [27]. Since EAT-2 is predominantly expressed by NK cells and human CD8^+^ T cells [9], it was important to determine whether there are additional binding partners for the phosphorylated tail of EAT-2 in human NK cells. We carried out an EAT-2 Y_127_ phosphopeptide pull-down of proteins using NK92 cell lysates. Proteins identified by at least 3 unique peptides associating with EAT-2 Y_127_ phosphopeptide but not with EAT-2 SH2 domain are listed in Table 5. Of these, only PLCγ1 and PLCγ2 are known to contain SH2 domains capable of phosphotyrosine-dependent interactions, and none of the proteins contain a Phospho-Tyrosine Binding (PTB) domain. In addition, PLCγ1 and PLCγ2 were identified with substantially more unique peptides and greater sequence coverage than any other proteins, suggesting lesser “hits” are likely to be non-specific. Notably absent from this list is Fyn, which we previously found to have a measurable affinity (K_D_∼1 μM) for the EAT-2 tail [22].

**Table 5:** Identification of proteins interacting with a phosphorylated, conserved motif in the tail of EAT-2.

Protein	Unique Peptides	Percent Coverage	SH2 Domain
**Phospholipase C gamma-1 (PLCγ1)**	**38**	**38.5**	**Y**
**Phospholipase C gamma-2 PLCγ2)**	**34**	**31.3**	**Y**
Prohibitin	4	24.3	N
Voltage-dependent anion-selective channel protein 1	3	21.6	N
3-ketoacyl-CoA thiolase, mitochondrial	5	20.4	N
Adenylate kinase 2, mitochondrial	3	18.0	N
EIF4A2	3	16.7	N
Transketolase	4	14.7	N
T-complex protein 1 subunit beta	5	12.0	N
CCT8	4	11.9	N
Bifunctional purine biosynthesis protein PURH	4	11.5	N
X-ray repair cross-complementing protein 5	3	10.7	N

Proteins identified by LC-MS/MS that associated with the phosphorylated tail peptide of EAT-2, but not with the SH2 domain are listed. PLCγ1 and PLCγ2 are the only SH2 domain-containing proteins listed, and show substantially greater sequence coverage than others listed.

**Figure 7:**
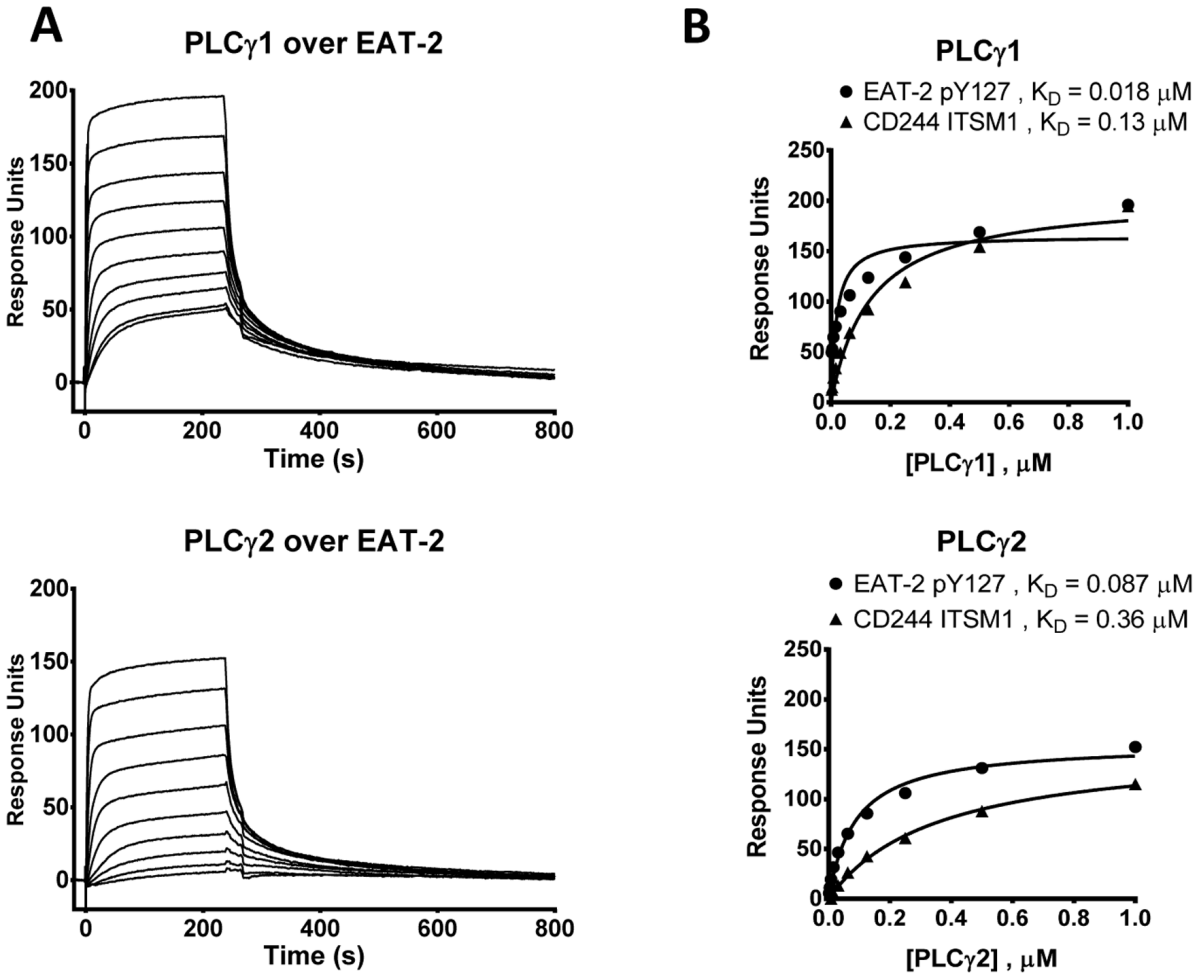
PLCγ1 and PLCγ2 bind the phosphorylated conserved motif in the tail of EAT-2. Overlaid SPR sensorgrams (A) and equilibrium binding curves (B) for ten serial injections of two-fold dilutions of human PLCγ1 SH2-SH2-SH3 or PLCγ2 SH2-SH2-SH3 (top concentration 1 μM) over immobilised phosphorylated peptides representing EAT-2 pY127, LAT pY132 or CD244 ITSM1 at 37°C. Signal from a blank reference cell was subtracted as background. K_D_ values were determined by fitting equilibrium binding data using a one-site specific binding model. Proteins were purified on the same day as SPR analysis.

We compared direct binding of PLCγ1 and PLCγ2 to EAT-2 Y_127_ and LAT Y_132_ by SPR. Direct binding of recombinant PLCγ1 to EAT-2 Y_127_ was confirmed (Figure 7 and Table 6 [27]). Recombinant PLCγ2 SH2 domains also bound immobilized EAT-2 Y_127_ and LAT Y_132_ peptides with comparable affinity (Figure 7, Table 6) demonstrating the ability of EAT-2 to recruit both PLCγ isoforms. PLCγ1 and PLCγ2 are encoded on human chromosomes 20 and 16 respectively and share only 63% sequence identity in their SH2 domains, so their overlapping specificity is for EAT-2 is remarkable. As for their relative affinity for EAT-2, we cannot rule out that the two fold lower affinities observed for PLCγ2 may be due to protein activity as recombinant PLCγ2 was not as stable as PLCγ1. Binding of CD244 ITSM1 to PLCγ was quantifiable but an order of magnitude weaker than the interaction between EAT-2 and PLCγ indicating a potential role for the adapter in linking surface receptors with effector enzymes (Figure 7, Table 6).

**Table 6:** Equilibrium dissociation constants for PLCγ1 and PLCγ2 binding phosphotyrosine motifs.

	PLCγ1 (SH2-SH2-SH3)			PLCγ2 (SH2-SH2-SH3)		
Peptide:	K_D_	range	*n*	K_D_	range	*n*
EAT-2 _Y127_	0.01	0.003–0.02	*2*	0.03	0.001–0.09	*3*
LAT _Y132_	0.02	0.01–0.02	*2*	0.02	–	*1*
CD244 ITSM1	0.8	0.13–1.5	*2*	0.21	0.06–0.36	*2*

Mean K_D_ values (μM) at 37°C for soluble recombinant proteins binding to immobilised phosphorylated peptides by SPR, range and number (n) of independent measurements. Proteins were purified on the same day as SPR analysis.

### SLAM family receptor mediated cytotoxicity is dependent on PLCγ1 and PLCγ2

**Figure 8:**
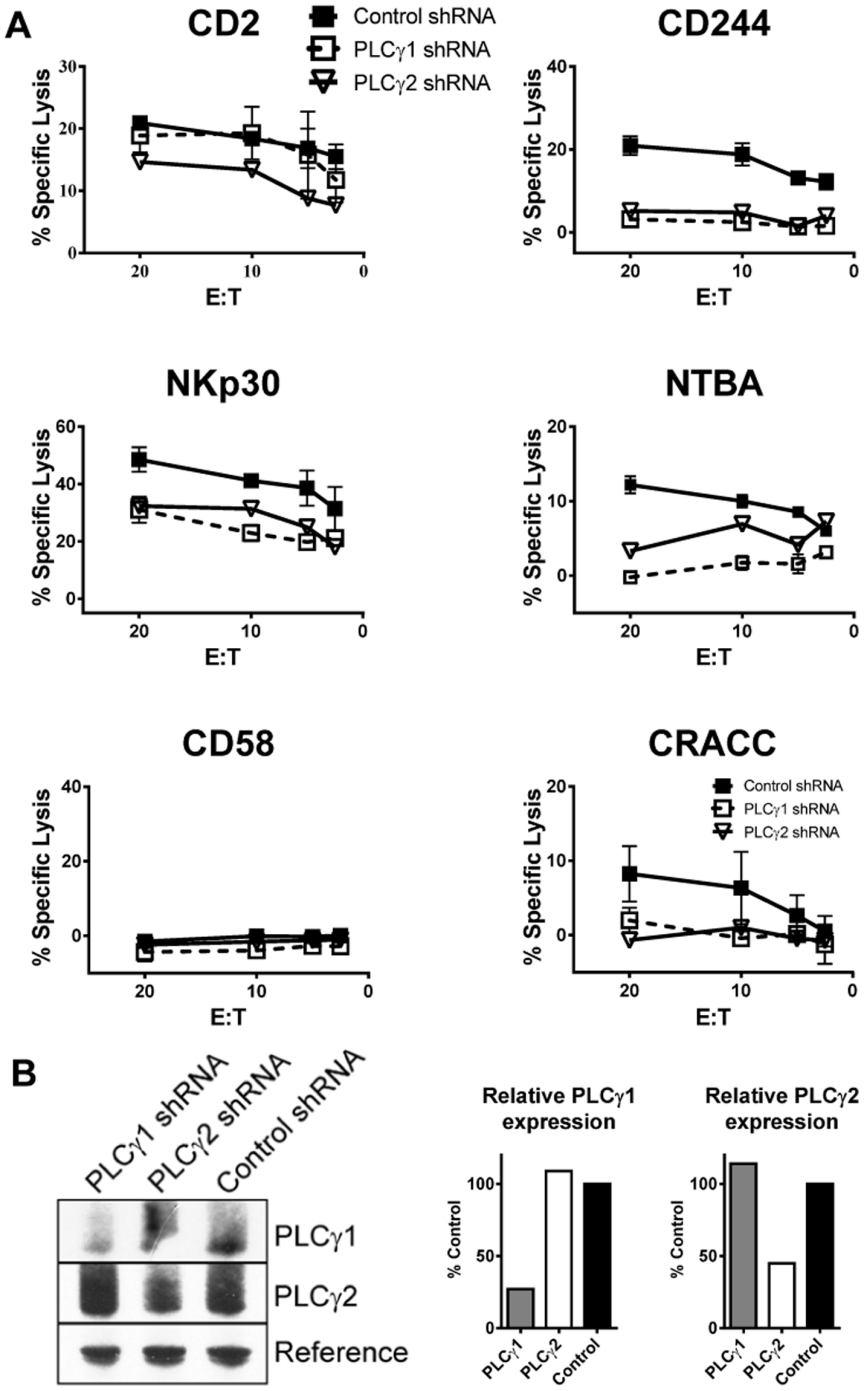
PLCγ1 and PLCγ2 are necessary for cytotoxicity through SLAM family receptors. (A) Redirected lysis of P815 cells by PLCγ1 or PLCγ2 knockdown in NK92 cells using the indicated activating antibodies. Error bars represent the mean +/− SEM of duplicate wells. Data are representative of 4 PLCγ1 and 2 PLCγ2 independent experiments. E:T = effector:target. (B) Quantitation of PLCγ1 and PLCγ2 expression in NK92 effector cells analysed by western blotting is shown in the bar graph. PLCγ1 was normalized to average lane intensity and PLCγ2 was normalized to the reference band in Odyssey.

PLCγ1 and PLCγ2 have distinct functions in NK cells [35]. PLCγ2 is expressed by NK cells and has been previously shown in mice to be critical for calcium flux and granule exocytosis during mouse NK cell activation [33]. Functional dependence of cytotoxicity on PLCγ1 has not been studied because mice lacking PLCγ1 are not viable. We compared the effects of knocking down PLCγ1 and PLCγ2 on SLAM family mediated cytotoxicity using lentiviral shRNA (Figure 8). Targeting of either PLCγ1 or PLCγ2 was isoform specific (Figure 8B). In the case of PLCγ1, protein expression was routinely reduced by 70% or more (Figure 8B). When PLCγ1 expression was suppressed, specific lysis of P815 cells through CD244, NTB-A and CRACC was reduced (Figure 8A). Consistent with a previous report that receptors that signal through ITAMs such as NKp30 can activate either PLCγ1 or PLCγ2 [49], a partial reduction in NKp30-mediated cytotoxicity in PLCγ1 knockdown cells was observed (Figure 8A). Consistent with data from PLCγ2^−/−^ mice [33], [35], [50], knockdown of PLCγ2 in human NK92 cells had a negative impact on cytotoxicity through all of the activating receptors analyzed. The data show clearly that SLAM family receptor mediated cytotoxicity depends on both isoforms PLCγ1 and PLCγ2.

## Discussion

Analysis of interactions of activating partners for ITSMs in the cytoplasmic regions of SLAM family receptors distinguished specificity of SAP and EAT-2 SH2 domains which correlates with published functional data [9], [30]. Independence of the SLAM family receptor, CRACC on SAP has been observed in SAP deficient mouse and human primary cells [9], [30]. However, we observed reliance of CRACC on SAP in redirected lysis assays in the human cell line, NK92. SPR analysis showed that SAP bound to human CRACC ITSM2, albeit two orders of magnitude more weakly than EAT-2, supporting the concept that there is competition between SAP and EAT-2 for SLAM family receptor ITSMs [9] depending on adapter concentration and differences in binding kinetics. The concentration of EAT-2 in NK92 cells was approximately 4 fold lower than that of SAP. However, it was two orders of magnitude above the K_D_ for binding ITSMs which may explain the marginal effects on cytotoxicity of halving the concentration of EAT-2 by RNA interference. Additional factors including phosphorylation of its tail are likely to be limiting in determining activating effects of EAT-2.

In our previous measurements of SH2 domain binding to phosphopeptides we observed a difference in specificity between tyrosine motifs which bound activating and inhibitory SH2 domains [41]. However, the potential of ITSMs to recruit inhibitory binding partners does not appear to involve an alteration in which motifs are likely to bind most strongly. There was a trend for the membrane proximal tandem ITSMs and ITIMs to bind more strongly, compared with the membrane distal ones, to the tandem SH2 domains of SHP-2 which may reflect some more general conservation of topology for multiple motifs [51]. Experiments with a recombinant cytoplasmic region of human CD244 have implicated CD244 ITSM3 as being dominant in SHP-1 binding [39] indicating other factors influence the relative importance of ITSMs in a given response.

Analysis of interactions of tandem SH2 domains of tyrosine phosphatases with tandem ITSMs indicated that there will be productive recruitment if concentrations of the adaptors, SAP and EAT-2 are compromised. This is consistent with functional data from SAP-family deficient mice [28] and human XLP patients. In the case of XLP patients, the function of NTB-A, which was mainly activating in NK clones from healthy individuals, became largely inhibitory in the absence of SAP. [18] Without SAP present, SH2 domains from SHP1 and SHP2 could more easily compete for ITSMs, and the likelihood of forming the higher avidity tandem interaction will increase.

There was a discernible difference between ITSMs and ITIMs in specificity for inhibitory binding partners. Subtle differences in sequence and consequent binding affinities may have a significant impact on signalling [52]. Increased affinity through tandem binding of SH2 domains to tandem phosphorylated tyrosine motifs over mono phosphorylated motifs has been observed previously and most likely contributes to the specificity of SH2 domain containing proteins [45], [53], though in our case we cannot exclude the contribution of additional sequence within the peptides, rather than a tandem domain interaction. Inhibitory effects of SLAM family receptors do not necessarily depend on tandem ITSM phosphorylation implying that single SH2 domain recruitment of inhibitory binding partners is feasible [37]. Activation of the single SH2 domain containing protein, SHIP is a proximal event in CD244 signal transduction [47] and there are data supporting competition between SAP and SHIP for a direct interaction with an ITSM in SLAM [40], [54]. A stronger dependency of mouse CD244 on SHIP compared with SHP-1 and SHP-2 [23] may reflect insufficient phosphorylation of tandem ITSMs. Alternatively, in mouse CD244, ITSMs 1 and 2 may be less favourably spaced for binding tandem SH2 domains, being further apart than in human CD244.

In contrast to ITSMs, the specificity of the conserved phosphotyrosine motif in the tail of EAT-2 appears to be more limited. We have characterized interactions with FYN and PLCγ, the latter binding up to two orders of magnitude more strongly [27]. In pulldown experiments by comparison with another phosphopeptide [27] or with the EAT-2 SH2 domain we identified both PLCγ proteins as being specifically isolated with the EAT-2 tail phosphopeptide, and PLCγ1 and PLCγ2 displayed similar specificity for EAT-2 and for the positive control phosphopeptide from LAT. Actual binding affinities in cells depending on phosphorylation of PLCγ may be an order of magnitude lower than measured by SPR with the recombinant PLCγ produced in bacteria [32]. Activation of PLCγ is a proximal event in SLAM family signal transduction [9], [31], [55] and we observed a dependence of cytotoxicity on both PLCγ1 and PLCγ2. We have observed a striking difference in the effects of PLCγ1 and PLCγ2 on IFNγ production by NK92 cells, inhibitory and activating respectively (TJW, unpublished) but the molecular mechanism of differences in regulation by the two isoforms is unknown [35]. Mechanisms of recruiting and activating PLCγ1 and PLCγ2 downstream of SLAM family receptors include colocalization of CD244 with LAT [55], [56]. A role for EAT-2 in activation of PLCγ is suggested by compromised calcium influx through CRACC in mice deficient in both EAT-2 and the mouse specific EAT-2 related protein, ERT [30]. Coprecipitation of EAT-2 and PLCγ1 in transduced hybridoma cells indicated this interaction could occur in cells [27]. Therefore recruitment of PLCγ by EAT-2 may provide an auxiliary mechanism for activating PLCγ.

In summary, we have identified differential and overlapping specificities of positive and negative regulators of cell signalling for the ITSMs of SLAM family receptors. The affinities measured indicate an intracellular environment where positive and negative regulators will compete for the phosphorylated ITSMs of SLAM family receptors. The relative affinities of the SH2 domains studied indicate that the adapters SAP and EAT-2 will play a dominant role in promoting cellular activation when present, however, signal attenuation by phosphatases is possible through direct recruitment to ITSM-containing receptors, especially in the absence of SAP and EAT-2.
